# Cardiovascular and metabolic characters of *KCNJ5* somatic mutations in primary aldosteronism

**DOI:** 10.3389/fendo.2023.1061704

**Published:** 2023-03-06

**Authors:** Yi-Yao Chang, Bo-Ching Lee, Zheng-Wei Chen, Cheng-Hsuan Tsai, Chin-Chen Chang, Che-Wei Liao, Chien-Ting Pan, Kang-Yung Peng, Chia-Hung Chou, Ching-Chu Lu, Vin-Cent Wu, Chi-Sheng Hung, Yen-Hung Lin, TAIPAI study group

**Affiliations:** ^1^ Cardiology Division of Cardiovascular Medical Center, Far Eastern Memorial Hospital, New Taipei City, Taiwan; ^2^ Graduate Institute of Medicine, Yuan Ze University, Taoyuan, Taiwan; ^3^ Department of Medical Imaging, National Taiwan University Hospital and National Taiwan University College of Medicine, Taipei, Taiwan; ^4^ Department of Internal Medicine, National Taiwan University Hospital Yun-Lin Branch, Yun-Lin, Taiwan; ^5^ Division of Cardiology, Department of Internal Medicine, National Taiwan University Hospital and National Taiwan University College of Medicine, Taipei, Taiwan; ^6^ Department of Medicine, National Taiwan University Cancer Center, Taipei, Taiwan; ^7^ Department of Obstetrics and Gynecology, National Taiwan University Hospital and National Taiwan University College of Medicine, Taipei, Taiwan; ^8^ Department of Nuclear Medicine, National Taiwan University Hospital, Taipei, Taiwan; ^9^ Division of Nephrology, Department of Internal Medicine, National Taiwan University Hospital and National Taiwan University College of Medicine, Taipei, Taiwan; ^10^ Cardiovascular Center, National Taiwan University Hospital, Taipei, Taiwan

**Keywords:** somatic mutation, *KCNJ5*, autonomous cortisol secretion (ACS), adrenocortical adenoma, cardiovascular system, metabolic syndrome

## Abstract

**Background:**

Primary aldosteronism (PA) is the leading cause of curable endocrine hypertension, which is associated with a higher risk of cardiovascular and metabolic insults compared to essential hypertension. Aldosterone-producing adenoma (APA) is a major cause of PA, which can be treated with adrenalectomy. Somatic mutations are the main pathogenesis of aldosterone overproduction in APA, of which *KCNJ5* somatic mutations are most common, especially in Asian countries. This article aimed to review the literature on the impacts of *KCNJ5* somatic mutations on systemic organ damage.

**Evidence acquisition:**

PubMed literature research using keywords combination, including “aldosterone-producing adenoma,” “somatic mutations,” “*KCNJ5*,” “organ damage,” “cardiovascular,” “diastolic function,” “metabolic syndrome,” “autonomous cortisol secretion,” etc.

**Results:**

APA patients with *KCNJ5* somatic mutations are generally younger, female, have higher aldosterone levels, lower potassium levels, larger tumor size, and higher hypertension cure rate after adrenalectomy. This review focuses on the cardiovascular and metabolic aspects of *KCNJ5* somatic mutations in APA patients, including left ventricular remodeling and diastolic function, abdominal aortic thickness and calcification, arterial stiffness, metabolic syndrome, abdominal adipose tissue, and correlation with autonomous cortisol secretion. Furthermore, we discuss modalities to differentiate the types of mutations before surgery.

**Conclusion:**

*KCNJ5* somatic mutations in patients with APA had higher left ventricular mass (LVM), more impaired diastolic function, thicker aortic wall, lower incidence of metabolic syndrome, and possibly a lower incidence of concurrent autonomous cortisol secretion, but better improvement in LVM, diastolic function, arterial stiffness, and aortic wall thickness after adrenalectomy compared to patients without *KCNJ5* mutations.

## Introduction

### Primary aldosteronism and its impact on the cardiovascular system and metabolic system

Primary aldosteronism (PA) was considered to be a rare disease but is now recognized to be the most common modifiable form of secondary hypertension (1–3). The prevalence ranges from 5% to 15% in hypertensive patients, and up to 20%-30% in those with resistant and refractory hypertension (4, 5). The two most common causes of PA are aldosterone-producing adenoma (APA), accounting for about 30-35% of all PA patients (6, 7) which can be treated by adrenalectomy, and bilateral adrenal hyperplasia (BAH), accounting for approximately 60-65% of patients with PA and is often treated with medications. The aldosterone overproduction in PA patients causes both cardiac structural changes, including left ventricular hypertrophy and remodeling (8), and declines in diastolic and systolic function (9). In addition, aldosterone-induced endothelial dysfunction plays an important role in vascular fibrosis and cellular hypertrophy, resulting in increased arterial stiffness (10–12). Clinically, PA is associated with higher cardiovascular morbidity and mortality, including stroke, coronary artery disease, atrial fibrillation, and heart failure, compared with essential hypertension (EH) (13–22). Moreover, aldosterone impairs glucose-stimulated insulin secretion and insulin sensitivity in skeletal muscle and adipocytes (23), which contributes to insulin resistance in humans (24). The prevalence of metabolic syndrome is also higher in PA patients compared to EH patients (25).

## Material and methods

We conducted a PubMed literature search, using a broad range of keywords combination, including “primary aldosteronism,” “aldosterone-producing adenoma,” “somatic mutations,” “pathogenesis,” “CYP11B2,” “*KCNJ5*,” “*ATP1A1*,” “*ATP2B3*,” “*CLCN2*,” “*CACNA1D*,” “*CACNA1H*,” “*CTNNB1*,” “organ damage,” “cardiovascular,” “cardiac,” “vascular,” “left ventricular,” “left ventricular mass,” “arterial stiffness,” “pulse wave velocity,” “diastolic function,” “metabolic syndrome,” “aortic wall,” “calcification,” “abdominal obesity,” “autonomous cortisol secretion,” “subclinical Cushing’s syndrome,” “adrenal vein sampling,” “NP59,” “steroid profiling.” We focused on various trials discussing the somatic mutations in APA and their impacts on clinical presentations published from 2011 to March 31, 2022. The retrieved articles were hand-selected according to the relevance cautiously. The reference lists of these selected articles were attentively reviewed to look for additional publications.

### The pathogenesis and types of somatic mutations in APA

The primary mineralocorticoid, aldosterone, is synthesized in the outer zone of the adrenal cortex, the zona glomerulosa (ZG) (26). In the ZG, 11-deoxycorticosterone is converted sequentially to corticosterone, 18-hydroxycorticosterone, and then aldosterone, catalyzed by the enzyme aldosterone synthase (encoded by the gene CYP11B2) (27). The production of aldosterone is normally regulated by angiotensin II (Ang II), serum potassium, and adrenocorticotropic hormone (ACTH) (28). However, the autonomous excess secretion of aldosterone in PA is independent of these factors.

**Figure 1:**
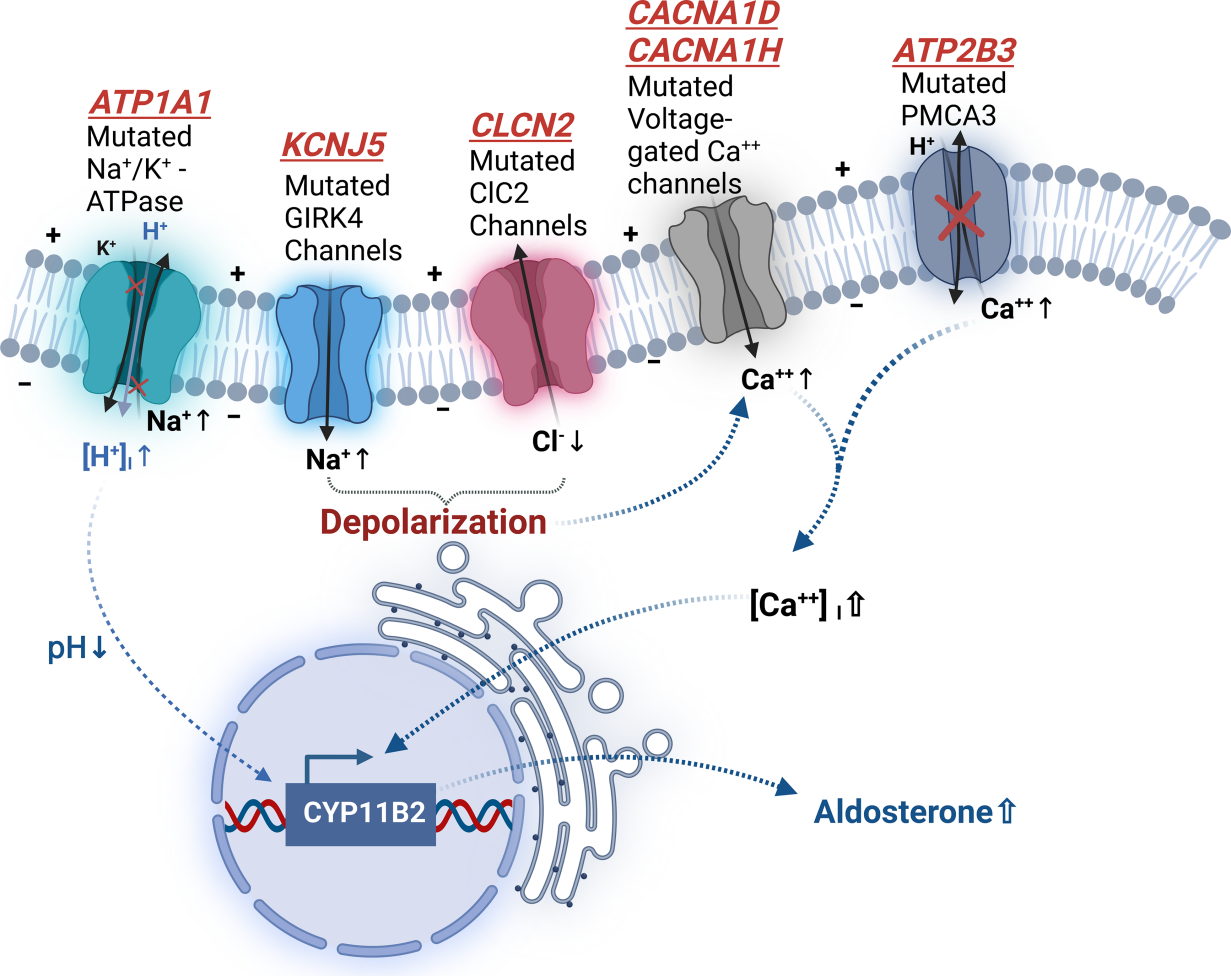
Somatic mutations related pathogenesis of aldosterone producing adenomas. *PMCA3: plasma membrane calcium-transporting ATPase 3.

In 2011, Lifton et al. were the first to report a somatic mutation in patients with APA (29). Somatic mutations occur in normal somatic cells, such as the adrenal gland, so that such mutations do not pass from parents to offspring. The location of the somatic mutation discovered by Lifton et al. is in the *KCNJ5* gene (Potassium Inwardly Rectifying Channel Subfamily J Member 5), which encodes G-protein-activated inward rectifier potassium channel (GIRK4). This potassium channel mediates the outward current of potassium ions to maintain hyperpolarization and stabilize resting membrane potential (30). However, mutations of the *KCNJ5* gene result in the channel losing its selectivity for potassium ions and increase the entry of extracellular sodium ions into the cell, causing the cell membrane depolarization, which causes the opening of voltage-gated calcium ion channel and allowing calcium ions to enter the cell. The increase in intracellular calcium ions induces transcription of the CYP11B2 gene through the activation of calcium signaling. Activation of aldosterone synthase causes excessive production of aldosterone and PA. Many somatic mutation sites of *KCNJ5* have now been identified, most of which can affect the selectivity of the GIRK4 channel (**Figure 1**), thus resulting in cell membrane potential depolarization, and ultimately increasing aldosterone production.

In addition, many other somatic mutations in APAs have been found to cause an increase in intracellular calcium ion concentration and result in aldosterone overproduction *via* various pathways, including *ATP2B3* (encoding the plasma membrane calcium-transporting ATPase 3, PMCA3) (31), *CLCN2* (encoding the chloride channel 2, ClC-2) (32), *CACNA1D* (encoding the voltage-gated calcium channels Cav1.3) (33) and *CACNA1H* (encoding Cav3.2) (34) genes (**Figure 1**). While the somatic mutations of *ATP1A1*(encoding the α1 subunit of Na^+^/K^+^ ATPase) genes result in increased intracellular Na^+^ concentration and H^+^ influx, which cause cells depolarized, but without significantly increased intracellular Ca^2+^ activity, and intracellular acidification which stimulate aldosterone production (31, 35). Mutations of *CTNNB1* gene (encoding β-catenin) have been shown to decrease the degradation of β-catenin, which causes an increase in the synthesis of aldosterone by regulating the WNT/β-catenin signaling pathway (36). In a recent study, double mutation of *CTNNB1* and *GNA11* (encoding the G protein subunit alpha11) or *GNAQ* (encoding the G protein subunit alpha q) results in upregulation of *LHCGR* (encoding the luteinizing hormone/choriogonadotropin receptor) and increase of aldosterone production (37).

### Prevalence of somatic mutations

The incidence of somatic mutations reported in a large European multicenter study of 474 patients with APA ranged from 27.2% to 56.8%, including 38% with *KCNJ5*, 9.3% with *CACNA1D*, 5.3% with *ATP1A1*, and 1.7% with *ATP2B3* mutations (38). In our previous study in Taiwan, we found that 128 of 219 (58.4%) patients with APA had somatic mutations, most of which were *KCNJ5* mutations (52.9%), followed by *CTNNB1* (3.7%), *ATP1A1* (1.4%), and *ATP2B3* (0.5%) (39). The expression of *KCNJ5* mutation differs in different ethnic groups. In Asia (Taiwan, Japan (40)) the prevalence of *KCNJ5* somatic mutations in APA patients is up to *55-75*%, compared to only about 25-50% in western countries (41). These data are mainly based on adrenal specimens from random biopsy and Sanger sequencing.

In recent years, with the progress in CYP11B2 immunohistochemical (IHC) staining-guided biopsy and next-generation gene sequencing (NGS), Rainey et al. reported that the detection rate of somatic mutations was nearly 90% (42). De Sousa et al. rechecked 14 specimens which were found to be negative for somatic mutations using traditional methods (random biopsy and Sanger sequencing) with CYP11B2-IHC staining-guided biopsy, and detected 11 more somatic mutations, thereby increasing the detection rate of somatic mutations from 71% (by traditional methods) to 94% (IHC-guided biopsy and NGS) (43). Interestingly, the newly detected mutations included 8 in the *CACN1D* gene, 2 in the *ATP1A1* gene, and 1 in the *ATP2B3* gene, but no new *KCNJ5* mutations were detected. In addition, they also found that *KCNJ5* somatic mutations could be detected in high, low, or even non-CYP11B2 IHC stained areas. These findings show that CYP11B2 staining-guided biopsy is an important tool to detect somatic mutations in PA patients, especially non-*KCNJ5* somatic mutations.

As shown in **Table 1**, the incidence of *KCNJ5* mutations in Asian (54, 55) populations is much higher than that in European (43) and American populations (42, 53), regardless of detection by traditional random biopsy with Sanger sequencing or by CYP11B2 IHC staining-guided biopsy and NGS. We previously found that *KCNJ5* somatic mutations were predominant in patients with APA harboring somatic mutations [*KCNJ5* accounts for 90.6% of APA patients with somatic mutations (39) detected by traditional methods, 81.2% of APA patients with somatic mutations by sequencing combined with Sanger and IHC-guided biopsy and NGS (55)]. Besides, the majority of studies investigating the clinical impacts of somatic mutations in APA patients are almost limited to comparing “*KCNJ5* mutations” with “non- *KCNJ5* mutations” (40, 41, 46, 48, 57–60). The clinical data of other somatic mutations in APA patients is rare. Therefore, in this article, we focus on the clinical impacts of *KCNJ5* somatic mutations in patients with APA.

**Table 1:** The detection rats of various somatic mutations in different countries dependent on traditional random biopsy or CYP11B2-IHC staining guided biopsy.

Country	Number	*KCNJ5*, %	*ATP1A1*, %	*ATP2B3*, %	*CACNA1D*, %	*CACNA1H*, %	*CTNNB1*, %	*CLCN2*, %	Non, %	Reference
Random biopsy + Sequencing										
French, Germany, Italy	308	38.3	5.2	1.6	NA	NA	NA	NA	55.3	(31)
USA	64	32.8	1.6	3.1	7.8	NA	7.8	NA	46.9	(33)
French, Germany, Italy	474	38	5.3	1.7	9.3	NA	NA	NA	45.7	(38)
Italy	112	39.3	6.3	0.9	NA	NA	NA	NA	53.5	(44)
USA, Germany	90	37.1	8.2	3.1	10.3	NA	2.1	NA	39.2	(45)
China	168	76.8	2.4	0.6	0.6	NA	NA	NA	19.6	(46)
Japan	108	69.4	2.8		1.9	NA	NA	NA	25.9	(40)
China	114	75.4	0	0	0.9	NA	NA	NA	23.7	(47)
Taiwan	148	59.5	1.4	0.7	0	NA	NA	NA	38.4	(48)
Japan	159	73.5	0.6	2.5	2.5	NA	NA	NA	20.8	(49)
Korea	66	71.2	0	0	0	NA	NA	NA	28.8	(50)
Taiwan	219	52.9	1.4	0.5	0	NA	3.7	NA	40.6	(39)
Japan	142	74.6	0.7	2.8	2.1	NA	0.7	NA	19.1	(51)
Brazil	100	43.4	2.6	1.3	NA	NA	2.6	NA	50.1	(52)
Addedly^*^ or purely identified by CYP11B2 IHC-guided biopsy + Sequencing										
USA	75	43	17	4	21	0	3	NA	12	(42)
USA	79	34	8	4	42	NA	0	NA	12	(53)
France^*^	48	42.9	12.2	10.2	26.5	0	0	0	6.1	(43)
Japan^*^	131	73	5	4	14	1	0	0	4	(54)
Taiwan^*^	240	63	2	1	4	2	5	1	22.5	(55)
Germany	41	56.1	12.2	4.9	9.8	0	0	2.4	14.6	(56)

NA, not available.

*Specimens detected by traditional random biopsy and Sanger sequencing. If there were no mutations detected, further CYP11B2-IHC guided biopsy and NGS would be applied for these specimens.

## *KCNJ5* somatic mutations

### Mutation sites of *KCNJ5*

The locations of most *KCNJ5* somatic mutations are within or near the potassium selectivity filter (among T149-G153) of the GIRK4 protein. The previously reported mutation sites in the literature are shown in **Table 2**.

**Table 2:** *KCNJ5* somatic mutations identified in APAs.

Mutations	Reference (First description)	Reported Year
p.Gly151Arg; Leu168Arg	Choi et al. (29)	2011
p.Ile157del	Murthy et al. (61)	2012
p.Glu145Gln	Åkerström et al. (62)	2012
p.Thr158Ala	Mulatero et al. (63)	2012
p.Glu145Lys	Azizan et al. (64)	2013
p.Thr149_Ile150insThr	Kuppusamy et al. (65)	2014
p.Trp126Arg	Williams et al. (44)	2014
p.Thr148_Thr149insArg	Zheng et al. (46)	2015
p.Arg115Trp; p.Glu246Gly	Cheng et al. (66)	2015
p.Ile157Lys; p.Phe154Cys; p.Ile150_Gly151insMet; p.Ile144_Glu145insAlaIle	Scholl et al. (45)	2015
p.Ala139_Phe142 dup	Hardege et al. (67)	2015
p.Glu147Gln_Thr149_Ile150insThrThrThr; p.Gly153_Gly164dup	Wang et al. (47)	2015
p.Thr148Ile; p.Thr149Ser	Nanba et al. (68)	2016
p.Glu145_Glu147delinsLys	Zheng et al. (69)	2017
p.Gly184Glu	Kitamoto et al. (51)	2018
p.Phe140Leu; p.Thr149delinsThrIle; p.Gly151_Tyr152del	Nanba et al. (42)	2018
p.Thr149delinsMetAla	Nanba et al. (53)	2019
p.Ile157_Glu159del	Peng et al. (70)	2021

### Basic clinical characteristics

In a meta-analysis study including 13 studies and 1636 patients, the patients with *KCNJ5* somatic mutations were significantly younger (45 ± 3 vs 52 ± 5 years), predominantly female (67% vs 44%), and had higher aldosterone level (42 ± 8 vs 33 ± 8 ng/dl), and larger tumor size (16.1 ± 6.4 versus 14.9 ± 7.4 mm) (71). In addition, lower potassium levels (38, 40, 48) and higher cure rates after adrenalectomy (48, 57) have also been reported in patients with *KCNJ5* somatic mutations in other studies compared to patients without *KCNJ5* mutations. Asian patients with *KCNJ5* somatic mutations have similar characteristics (48) but without differences in sex and tumor size compared to patients without *KCNJ5* somatic mutations (39, 46, 50, 72). Due to differences in the incidence of *KCNJ5* somatic mutations between Eastern and Western populations, the cure rate of hypertension post-adrenalectomy may also be affected by ethnicity. Investigations of the impacts of *KCNJ5* mutations on symptoms, prognosis, and even systemic target organ damage, including cardiovascular structure and function, and metabolic disorders, may affect the treatment strategy of patients.

### Relationships of *KCNJ5* somatic mutations with cardiac structure and function

#### Left ventricular mass

In animal studies, excessive aldosterone with salt intake has been shown to increase bilateral ventricular fibrosis (73–75) and left ventricular hypertrophy (73, 75). Clinical studies have also revealed that patients with PA have higher rates of cardiac fibrosis and left ventricular hypertrophy than patients with EH (76–78). LVM can be roughly divided into predicted LVM (pLVM) (79–81) and inappropriately excessive LVM (ieLVM) (80, 82–84), denoting the hemodynamic and non-hemodynamic contributions to LVM, respectively. Inappropriate LVM has been shown to be higher in APA patients compared with EH patients and to decrease after adrenalectomy in APA patients (82).

Only some studies have discussed the impact of *KCNJ5* somatic mutations on the left ventricular structure. Rossi et al. (41) were the first to report that APA patients with *KCNJ5* mutations had a higher LVM index (LVMI) than patients without mutations, even with a higher ratio of female patients. A similar trend albeit without statistical significance was also noted in another study in China, in which patients with *KCNJ5* mutations had higher baseline systolic blood pressure, which may have led to an increase in LVMI (47). Interestingly, other studies have not shown a difference in LVMI between patients with and without mutations (40, 46, 48). This discrepancy may be due to differences in age, sex, hypertension duration, or the number of antihypertensive drugs between the studies, which may have influenced LVM.

In our previous study, after matching for age, sex, body mass index (BMI), and hypertension status, *KCNJ5* mutation carriers had a higher aldosterone level, LVMI, and inappropriately excessive LVMI (ieLVMI) than non-carriers (57). We also found that the increased LVMI in *KCNJ5* mutation carriers was mainly attributable to ieLVMI *via* a non-hemodynamic pathway, thus probably caused by a higher aldosterone level (57). Furthermore, the decreases in LVMI and ieLVMI after adrenalectomy were higher in *KCNJ5* mutation carriers than in non-carriers (57).

In short, the impact of *KCNJ5* somatic mutations on LVMI seems to be discrepant in different studies, which may relate to different patients’ baseline characteristics. However, after matching age, sex, and blood pressure status, the APA patients with *KCNJ5* somatic mutations seem to have higher LVMI, ieLVMI, and greater improvement after adrenalectomy.

#### Diastolic function

Excessive aldosterone results in cardiac fibrosis and left ventricular hypertrophy (76–78), which contribute to impaired left ventricular relaxation in patients with PA (85–87). However, few studies have discussed left ventricular diastolic function in patients with *KCNJ5* mutations. Rossi et al. (41) reported no difference between patients with and without *KCNJ5* mutations in atrial contribution to left ventricular filling (ACLVF) or E/e’, and only ACLVF was significantly lower in the patients with *KCNJ5* mutations, but not in those without mutations after adrenalectomy. In another study (57), only e’ was significantly higher in *KCNJ5* mutation carriers compared to non-carriers before surgery, even after matching for age, sex, and hypertension status between both groups. After adrenalectomy, a significant decrease in E/e’ and a borderline increase in e’ were noted in *KCNJ5* mutation carriers, but not in non-carriers (57).

In short, patients with *KCNJ5* mutations benefit from adrenalectomy, not only in the left ventricular structure but also in diastolic function.

### Relationships of *KCNJ5* somatic mutations with vascular structure and function

#### Thickness and calcification of the aorta

PA is associated with increased intima-media thickness of the carotid artery (88, 89). A recent study demonstrated that APA patients with *KCNJ5* mutations had a thicker abdominal aorta, but less abdominal aorta calcification compared to patients without mutations on abdominal CT (58). Moreover, patients harboring *KCNJ5* mutations had greater improvements in abdominal aorta thickness compared to those without mutations after adrenalectomy (58).

#### Arterial stiffness

Aldosterone infusion accompanied by a salty diet was shown to increase arterial stiffness and fibronectin accumulation in an animal study, which was independent of normotensive controls and reversed by an aldosterone antagonist (90). Clinically, arterial stiffness can be evaluated by pulse wave velocity (PWV). Previous studies have reported higher PWV in PA patients compared with EH patients (91). In addition, APA patients with *KCNJ5* mutations have been shown to have lower PWV compared to those without mutations (40, 48). However, the patients with mutations were younger in these studies, which may have resulted in a lower PWV and interfered with the effect of *KCNJ5* mutations. In another study comparing patients with and without *KCNJ5* mutations matched for age, sex, and BMI, there was no difference in PWV between the two groups. However, there was a trend of a greater decrease in PWV after adrenalectomy in the patients with *KCNJ5* mutations (59).

A recent study (60) revealed lower brachial-ankle PWV (baPWV) in patients with *KCNJ5* mutations compared to those without mutations before propensity score matching (PSM), but similar baPWV in both patients with and without mutations after matching for age, sex, BMI and hypertension status. After adrenalectomy, the decrease in baPWV in APA patients with *KCNJ5* mutations was greater than that in those without mutations both before and after PSM. Furthermore, only the APA patients with *KCNJ5* mutations had a significant decrease in baPWV after adrenalectomy, which was not found in the patients without mutations either before or after PSM. The patients with *KCNJ5* mutations were correlated with a change in baPWV even after adjusting for age, sex, and hypertension status both before and after PSM. The possible causes of the greater decrease in PWV after surgery in *KCNJ5* mutation carriers may be due to higher baseline aldosterone levels, less residual hypertension, and lower incidence of autonomous cortisol secretion (92) compared to patients without *KCNJ5* mutations.

In short, with comparable age, sex, and hypertension status, there is no difference in arterial stiffness between patients with and without *KCNJ5* mutations, but patients with *KCNJ5* mutations have a greater improvement in arterial stiffness after adrenalectomy.

### Relationships of *KCNJ5* somatic mutations with metabolic disorder and abdominal obesity

Metabolic syndrome (MetS) is a combination of metabolic abnormalities, including obesity, diabetes, dyslipidemia, and hypertension (93). In spite of the considerable amount of previous studies that have revealed that excessive aldosterone is related to the development of MetS (94, 95), a large controlled cross-sectional study has shown no significant difference in metabolic profiles between PA and EH patients (96). Various diagnosis criteria of MetS and ratios of unilateral or bilateral PA in different studies may cause heterogeneous prevalence of MetS in patients with PA (97).

Although APA leads to higher aldosterone secretion compared to BAH (98), several reports have revealed higher prevalence rates of MetS, obesity, and dyslipidemia in patients with BAH compared to those with APA (97, 99–101). Youichi et al. found that after adjusting background characteristics, including PAC, patients with BAH still have a higher prevalence of obesity than patients with APA (100). These findings suggest PAC may be not the only contributor to metabolic disorders in PA patients. Therefore, obesity itself may be the potential contributor to the higher prevalence of MetS in BAH. The relevance between aldosterone overproduction and obesity is vague in patients with APA and BAH and further investigations were needed.

CT is a well-established imaging tool to quantify abdominal adipose tissue, utilizing the Hounsfield Units (HU) range to measure the subcutaneous and visceral fat areas (102). Concerning the effect of *KCNJ5* somatic mutations on MetS, Chen et al. first reported that APA patients with *KCNJ5* mutations had fewer MetS, lower triglyceride (TG) levels, waist circumference, and subcutaneous adipose tissue (SAT) and visceral adipose tissue (VAT) area than those without *KCNJ5* mutations even after matching for age (103). APA patients with *KCNJ5* mutations also had fewer MetS and lower triglyceride levels compared to patients with BAH. Furthermore, APA patients with *KCNJ5* mutations have been reported to have significantly increased abdominal adipose tissue after adrenalectomy, but not in patients without mutations (103). A previous study has reported that aldosterone and mineralocorticoid receptors play an important role in adipose tissue development (104). An increase in adipose tissue area after adrenalectomy was reported in APA patients (105). However, the mechanism of these findings is still uncertain and may be intertwined with many factors in addition to excessive aldosterone.

The evidence about the direct effect of aldosterone on lipid metabolism has not been confirmed. Some studies reported lipid disorders were similar in patients with PA and EH (25, 106), but others showed a positive correlation between aldosterone and TG and low-density lipoprotein (107, 108). Interestingly, deterioration in lipid metabolism has been found in APA patients after adrenalectomy (105, 109). This change may be driven by the decline of renal function after treatment of PA, which would lead to a decrease of lipoprotein lipase and hepatic triglyceride lipase and interfere with the metabolism of lipids (110). As for *KCNJ5* mutation, the previous study revealed only patients with mutations had a significant increase of TG after adrenalectomy, but not in patients without mutations (103).

In short, APA patients appear lean, especially those with *KCNJ5* mutations, but physicians should be aware of the high risk of cardiovascular diseases related to aldosterone toxicity and worsening dyslipidemia after adrenalectomy (103). However, considering the complex interaction of aldosterone and MetS, and the only study discussing the impacts of *KCNJ5* mutations on MetS, further investigation is necessary.

### Relationship of *KCNJ5* somatic mutations with subclinical hypercortisolism

Autonomous cortisol secretion (ACS), formerly known as subclinical Cushing syndrome or subclinical hypercortisolism, is characterized by autonomous cortisol hypersecretion from adrenal adenomas or hyperplasia, but the absence of clinical symptoms of overt Cushing’s syndrome (111–113). The prevalence of ACS has been reported to be around 30% in patients with adrenal incidentalomas (113), and 12.8% to 32% in patients with concurrent ACS and PA (92, 114–116). Patients with ACS have a higher risk of hypertension, obesity, dyslipidemia, hyperglycemia, adverse cardiovascular events, and mortality compared to patients with nonfunctional adrenal tumors (113, 117). PA patients with concurrent ACS have also been reported to have a higher incidence of cardiovascular and metabolic complications than patients with pure PA (118–122).

Recently, *KCNJ5* mutations have been identified in some aldosterone- and cortisol-co-secreting adrenal adenomas (123). Interestingly, results from the TAIPAI study group indicated that ACS (1 mg dexamethasone suppression test > 1.5 µg/dL) is more common in APA patients without *KCNJ5* mutations than in patients with *KCNJ5* mutations (92). Furthermore, APA patients without *KCNJ5* mutations and concurrent ACS were shown to have a lower clinical success rate (36.8%) after adrenalectomy compared to patients with *KCNJ5* mutations and concurrent ACS (42.9%) (92).

The IHC examination of CYP11B2 and CYP11B1, key enzymes in aldosterone and cortisol biosynthesis, in adrenal slices is commonly used to identify the source of aberrant secretions of aldosterone and cortisol (124, 125). A recent study demonstrated that the immunoreactivity of CYP11B1 was higher in adrenal adenomas of PA patients with concurrent ACS compared to PA patients without ACS (126). Interestingly, some studies have reported that the immunoreactivity of CYP11B1 was higher in adenomas without *KCNJ5* mutations compared with adenomas harboring mutant *KCNJ5 (* 92, 127). These results seem to support that patients without *KCNJ5* mutations are more likely to have concurrent ACS than patients with adenomas harboring mutant *KCNJ*5. However, other studies showed that the immunoreactivity of CYP11B1 was relatively low in adenomas without *KCNJ5* mutations (43, 128). Therefore, further research is still needed to explore whether *KCNJ5* mutations can directly or indirectly affect the occurrence of ACS.

### Pre-operative differentiation of mutations

Somatic mutations in APA patients can only be detected using adrenal gland specimens after surgery. However, this is not helpful to predict the cure rate or long-term prognosis before surgery. Although adrenalectomy currently is preferred for patients with PA concerning the risk of all-cause mortality and major adverse cardiovascular events compared to medical treatment (129), predicting the types of mutations with simple and safe methods before surgery would possibly allow physicians to make a comprehensive treatment strategy for patients. Future studies are needed to explore the most appropriate diagnostic methods.

#### 18-oxocortisol, 18-hydroxycortisol, and other steroid fingerprints to predict KCNJ5 mutations

Steroid profiling using tandem mass spectrometry has shown promising results for the pre-operative differentiation of somatic mutations. APA patients with *KCNJ5* mutations have been shown to have a distinct steroid signature, with the highest concentrations of 18-hydroxycortisol and 18-oxocortisol in the plasma from both adrenal and peripheral veins (130). A comprehensive mass spectrometry imaging study also reported elevated intensities of 18-hydroxycortisol and 18-oxocortisol in *KCNJ5*-mutated APA specimens (127). A study utilizing a steroid panel consisting of aldosterone, 18-hydroxycortisol, 18-oxocortisol, 11-deoxycorticosterone, corticosterone, cortisol, and 21-deoxycortisol in plasma from peripheral veins showed that 92% of APAs could be classified according to their underlying somatic mutations (130).

#### Adrenal vein sampling to predict KCNJ5 mutations

Adrenal vein sampling is the gold-standard diagnostic procedure for the identification of surgically curable patients with PA. Conflicting results have been reported regarding the relationship between somatic mutations of PA and adrenal vein sampling results. Seccia et al. reported a higher lateralization index in PA patients with *KCNJ5* mutations, probably due to higher aldosterone secretion (131). In contrast, another study showed that the lateralization index between mutation non-carriers, ATPase-mutated, and *KCNJ5* mutated patients was not significantly different (132). These discrepant findings may be due to the different lateralization indexes used (2 and 4 respectively) impeding direct comparisons of the two studies.

#### NP-59 to predict KCNJ5 mutations

NP-59 adrenal scintigraphy is a functional study used to evaluate adrenal cortical activity. Lu et al. reported using semiquantitative NP-59 adrenal scintigraphy as an imaging biomarker to predict *KCNJ5* mutations in PA patients (133). Among 62 PA patients who underwent NP-59 adrenal scintigraphy with available *KCNJ5* mutation status, adrenal-to-liver ratio (ALR) and maximal count ratio between two adrenal glands (contrast, CON) derived from NP-59 adrenal scintigraphy were used to differentiate patients with and without *KCNJ5* mutations. The results showed that the patients with *KCNJ5* mutations had significantly higher ALR and CON compared to those without *KCNJ5* mutations. Using optimal cutoff values of ALR and CON, NP-59 adrenal scintigraphy could predict *KCNJ5* mutations with sensitivity and specificity of 85% and 57%, respectively. This is the first study using single photon emission computed tomography (SPECT) to predict somatic mutation status in PA patients.

## Limitations

There are two major limitations in this review. First, in most of the studies discussing the cardiovascular or metabolic impacts of *KCNJ5* somatic mutations in APA patients, the mutation detection was made by traditional methods (random biopsy and Sanger sequencing). The group called “non-*KCNJ5* mutations” were not homogeneous, that might induce a bias in the interpretation of the results. As the advance of genotype detection improved, further studies discussing the difference of cardiovascular or metabolic impacts among various somatic mutations are expected. Second, the majority of the studies analyzing the impacts of *KCNJ5* somatic mutations on cardiovascular or metabolism were performed in Asian cohorts. Since the prevalence of *KCNJ5* somatic mutations in Asia is higher than in other western countries, the ethnic and environmental factors may involve in the difference observed among APA patients with or without *KCNJ5* mutations. We look forward to the clinical outcome data of *KCNJ5* somatic mutations from other countries other than Asia.

## Prospects

*KCNJ5* somatic mutations have been shown to be a good prognostic predictor for the remission of hypertension after unilateral adrenalectomy in APA patients (52), and steroid profiling to predict mutation status may be of value to make a comprehensive plan of treatments. However, more studies are still needed to validate the diagnostic value of steroid profiling in APA patients of different ethnicity.

Mutated potassium channel GIRK4, coded by a mutated *KCNJ5* gene, has been demonstrated to have different pharmacological characteristics to the wild-type channel. The calcium channel blocker verapamil and macrolides such as amiloride have shown particularly strong inhibitive abilities for mutant channels (134, 135). Targeted blockade of mutated GIRK4 may offer new therapeutic strategies for APA patients with *KCNJ5* mutations who are unsuitable for surgery.

**Figure 2:**
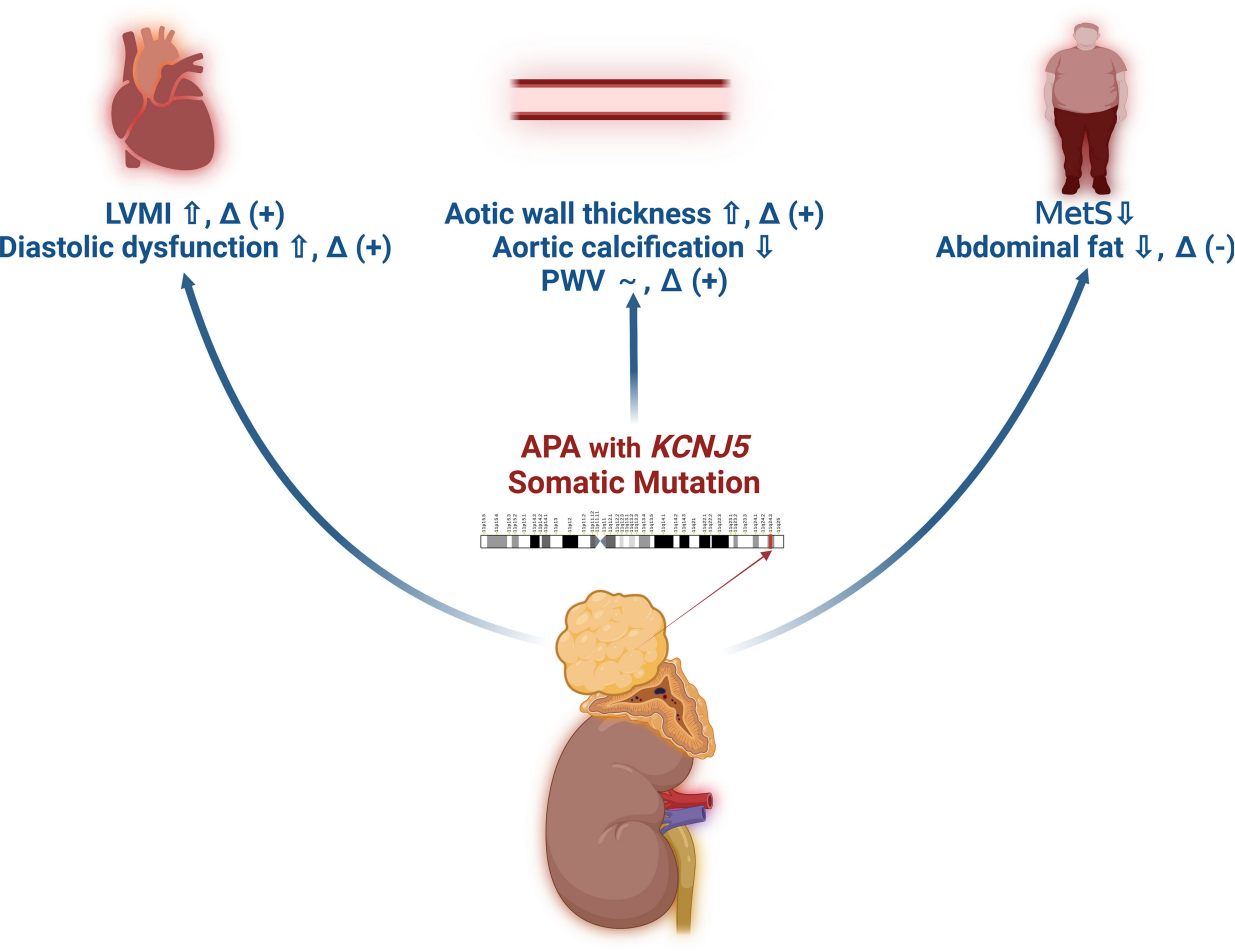
The schematic diagram of the effect of *KCNJ5* somatic mutations on target organ damage in patients with aldosterone producing adenoma. *~: similar results between patients with or without *KCNJ5* somatic mutations. *Δ (+): Improvement after adrenalectomy. *Δ (-): Deterioration after adrenalectomy.

In conclusion, *KCNJ5* somatic mutations in patients with APA play an important role in cardiovascular outcomes (**Figure 2**), including higher LVM, more impaired diastolic function, thicker aortic wall, lower incidence of MetS, and possibly a lower incidence of concurrent ACS, but better improvement in LVM, diastolic function, arterial stiffness, and aortic wall thickness after adrenalectomy compared to patients without *KCNJ5* mutations.

## Author contributions

Y-YC: Project concept and design, studies review (CV outcomes), initial draft writing. B-CL: Data collection and editing (aorta aspects). Z-WC: Data collection and editing (background). C-HT: Data collection and editing (mechanism). C-CC: Data collection and editing (metabolic aspects). C-WL: Critical revision. C-TP: Critical revision. K-YP: Critical revision and data collection and editing (gene detection). C-HC: Critical revision. C-CL: Data collection and editing (nuclear medicine). V-CW: Project concept and data collection. C-SH: Critical revision. Y-HL: Project concept and design, data collection, and critical revision. All authors contributed to the article and approved the submitted version.

## Appendix

Membership of the Taiwan Primary Aldosteronism Investigation (TAIPAI) Study Group: Che-Hsiung Wu, MD (Chi-Taz Hospital,PI of Committee); V-CW, MD (NTUH, PI of Committee); Y-HL, MD (NTUH, PI of Committee); Hung-Wei Chang, MD, PhD (Far Eastern Clinics, PI of Committee); Lian-Yu Lin MD, PhD (NTUH, PI of Committee); Fu-Chang Hu, MS, ScD, (Harvard Statistics, Site Investigator); Kao-Lang Liu, MD (NTUH, PI of Committee); Shuo-Meng Wang, MD (NTUH, PI of Committee); Kuo-How Huang, MD (NTUH, PI of Committee); Yung-Ming Chen, MD (NTUH,PI of Committee); C-CC, MD (NTUH, PI of Committee); Shih-Cheng Liao, MD (NTUH, PI of Committee); Ruoh-Fang Yen, MD,PhD (NTUH, PI of Committee); and Kwan-Dun Wu, MD, PhD (NTUH, Director of Coordinating Center).
